# Physico-Chemical Properties and Nutrition of Marine Lipids

**DOI:** 10.3390/foods12224078

**Published:** 2023-11-10

**Authors:** Eva Falch

**Affiliations:** Department of Biotechnology and Food Science, NTNU—Norwegian University of Science and Technology, P.O. Box 8900, NO-7491 Trondheim, Norway; eva.falch@ntnu.no; Tel.: +47-930-98888

## 1. Introduction

Marine lipids, especially long-chain polyunsaturated omega-3 fatty acids, play important roles in human nutrition and health. Despite their well-documented health effects, the global population generally do not reach these recommended beneficial levels through the consumption of seafood or dietary supplements. It has been suggested that only 20% of the world population consumes the minimum amounts of marine lipids required recommended for health benefits [1]. Today, the main dietary sources of marine lipids are fatty fish, such as anchoveta, tuna, herring, sardines, mackerel, salmon, trout, and fish oils, from sources like anchoveta, sardines and cod liver. Moreover, shellfish, such as crab, mussels, and oysters, act as lipid sources in human diets. According to Food and Agriculture Organization [2], the global marine database includes catches of more than 2600 species, and many of these species are not being fully exploited for their potential as dietary marine lipid sources for food and health. These can be species found at lower tropic levels than those caught for commercial fisheries, and they may become important for future food security and nutrition. Additionally, seafood resources that are lost through the value chain may be upcycled as marine lipid sources and reintroduced into the food value chain as food for human consumption or a lipid source for feed. Marine lipids are scarce resources, and these new unexplored or underutilized marine biomasses may become a nutrient source for the growing world population. For this to happen, more knowledge is needed to explore what marine lipids the ocean can provide and their nutritional and chemical composition, stability, and physico-chemical properties to uncover their potential applications in future products. It is important to understand the nature of the properties of marine lipids during their storage, handling, processing, and fortification into food or feed products in order to improve their potential use as ingredients. This Special Issue conducts research to increase the availability and consumption of marine lipids. This could include studies of new and current marine lipid resources and their properties in food, consumption and stability, logistics and value chains, extraction and refinement, technologies, and consumers’ insights regarding marine lipids in food. Food production leads to the depletion of our natural resources [3], and there are already supply challenges in terms of preparing for and securing future nutrition in a sustainable way. It is expected that a significant portion of our food supply will have to be sourced from the ocean. According to the Global Nutrition Report [3,4], poor diets and malnutrition are still among the greatest global social challenges of our time, and research into how marine lipids can contribute to overcoming this problem is important.

## 2. Composition of Marine Lipids

Marine lipids comprise a variety of lipid classes and fatty acids with different properties. The major marine lipid constituents are triacylglycerols, comprising more than 95% of food lipids, followed by phospholipids and sterols. There are also minor lipid components, such as carotenoids, vitamins, sphingolipids, sterol esters, wax esters, free fatty acids, and partial acylglycerols. Fatty acids are the main constituents of several lipid classes, in particular acylglycerols and phospholipids, and these fatty acids greatly define their functionality, stability, and health properties. The most known marine fatty acids are the long-chain polyunsaturated omega-3 fatty acids (LC-PUFA) eicosapentaenoic acid (EPA 20:5 n-3) and docosahexaenoic acid (DHA 22:6 n-3), as well as docosapentaenoic acid (DPA 22:5 n-3), which are considered to be part of the long-chain polyunsaturated omega-3 fatty acid group. EPA and DHA are only naturally found in the marine environment. There are also 10-fold more fatty acids arranged in the lipid molecules, making up a range of different fatty acid combinations, lipid molecules, and functionalities. The lipid and fatty acid composition may vary based on the species, organs, gender, age, maturity stage, feed, temperature, etc. Moreover, the compositions of lipids in membranes and storage might depend on the temperature, and lower temperatures are associated with increased unsaturation, which maintains membrane fluidity and functionality at lower temperatures [5]. The feed is also of high importance, since it is reflected in the organism’s organs. Fish do not usually synthesize the LC-PUFA omega-3 fatty acids, which originate from phytoplanktons and algae that accumulate along the tropic chain. There are still knowledge gaps regarding different sources of marine lipids and their compositions, and it is important to further explore new and potential food resources for improving the health of the growing population, sustainably harvesting the ocean’s food resources, and securing the ocean ecosystems.

## 3. Health and Nutrition

Marine lipids have been in commercial use for their health benefits since 1780, when cod liver oil was used as a supplement for providing vitamins A and D. This was long before the term long-chain polyunsaturated omega-3 fatty acids became a topic of scientific interest. In the 1970, the cardioprotective effects of these fatty acids were discovered through studies of Greenland Eskimos. This discovery was followed by better documentation of cardiovascular effects, inflammation, and brain development. Today, the substantiated daily levels of EPA + DHA range from 250 to 500 mg/per day for the maintenance of normal cardiac function, brain function, and vision, while higher concentrations are recommended for the maintenance of normal blood pressure and blood concentrations of triacylglycerols [6,7,8]. Coronary vascular disease is the leading cause of death worldwide [9], and strategies to increase the intake of these potent fatty acids would make a major contribution to public health and wellbeing. Understanding the bioaccessibility, extraction, stability, and bioavailability of different marine lipids and understanding consumers and their food choices are key elements in such strategies.

## 4. Physico-Chemical Properties

The physico-chemical properties of marine lipids play crucial roles in the quality and behavior of the lipids during the processing and designing of food, feed, or dietary supplements. These properties usually include (1) the crystallization and melting properties; (2) the hydrophobic, hydrophilic, and interfacial properties affecting emulsification; (3) rheological properties such as flow and viscosity; (4) oxidative stability; (5) color and optical properties; and (6) metabolic properties, etc. Moreover, lipid droplet size, structures within the lipid molecules, and three-dimensional structures may also play important roles in determining the physico-chemical properties. These properties vary due to the lipid types, such as the combination of fatty acids and their degree of unsaturation, triacylglycerols, phospholipids, and other lipid molecules. 

The lipid class composition in the raw material will affect the possibility of isolating and extracting the lipids on an industrial scale. While raw materials comprising phospholipids can be easily extracted on a small scale in a laboratory using strong chemicals, industrial extraction using green technologies is more challenging due to solubility issues and may result in lower lipid yield, favoring certain lipid molecules and giving non-representative lipid isolates.

Marine oils for human consumption are usually refined, purified, or further processed into omega-3 concentrates. The physico-chemical properties play a major role in many of these refinement steps, and they are the principle behind molecular distillation (boiling properties and volatility), alkali refinement (saponification), and winterization (crystallization properties). Consequently, the lipids’ physico-chemical properties not only change due to this processing stage but also result in several lipid side streams with potential usage. 

Another important factor affecting the functionality is biochemical degradation, such as autolysis, which changes the lipid molecules. This can be the action of endogenous enzymes like (1) lipases that catalyze the hydrolyzation of triacylglycerols into partial acylglycerols and free fatty acids; (2) phospholipases that hydrolyze phospholipids into lysophospholipids, free fatty acids, and other smaller molecules; and (3) other enzymes that generate larger molecules through processes such as esterifying the cholesterols into cholesteryl esters. All these reactions will result in the formation of new lipid molecules via a change in the physico-chemical properties, thereby acting differently during processing and product formulations. Lipid oxidation is another challenge that needs full attention during the whole value chain from raw materials to consumer-ready products. Encapsulation is often used to protect marine lipids from oxidizing and as a functional carrier of lipid nutrients. Microencapsulation is one way of protecting the oil against lipid oxidation in the food matrix. 

Exploring these properties is essential for understanding the interplay between molecules during the whole value chain, leading to optimized product formulations that meet the consumer’s need. Finally, more research is needed to better understand future marine lipid sources and how these can be used in products with benefits for human nutrition and health. 
